# Assessing Biocompatibility of Composite Cements by Peri/Intramuscular and Subcutaneous Implantation in Rats

**DOI:** 10.3390/biomedicines12081718

**Published:** 2024-08-01

**Authors:** Alina Ioana Ardelean, Sorin Marian Marza, Andrada Negoescu, Madalina Florina Dragomir, Codruta Sarosi, Marioara Moldovan, Razvan Ene, Liviu Oana

**Affiliations:** 1 Department of Veterinary Surgery, Faculty of Veterinary Medicine, University of Agricultura Sciencies and Veterinary Medicine, 400372 Cluj-Napoca, Romania; alina-ioana.ardelean@usamvcluj.ro (A.I.A.); madalina.dragomir@usamvcluj.ro (M.F.D.); oanaliviu2008@yahoo.com (L.O.); 2 Department of Veterinary Imagistics, Faculty of Veterinary Medicine, University of Agricultura Sciencies and Veterinary Medicine, 400372 Cluj-Napoca, Romania; 3 Department of Veterinary Pathology, Faculty of Veterinary Medicine, University of Agricultura Sciencies and Veterinary Medicine, 400372 Cluj-Napoca, Romania; andrada.negoescu@usamvcluj.ro; 4 Department Polymeric Composites, Babeș-Bolyai University, 400294 Cluj-Napoca, Romania; liana.sarosi@ubbcluj.ro (C.S.); marioara.moldovan@ubbcluj.ro (M.M.); 5 Department Orthopedics, Anesthesia and Intensive Care, University of Medicine and Pharmacy “Carol Davila”, 020021 Bucharest, Romania; 6 Orthopedics and Traumatology Department, Bucharest Emergency University Hospital, 050098 Bucharest, Romania

**Keywords:** biocompatibility, composite cements, subcutaneous, peri/intramuscular, rats

## Abstract

This study’s goal was to evaluate the biocompatibility of two composite cements over a 90-day period by analyzing the individuals’ behavior as well as conducting macroscopic and histological examinations and Computed Tomography (CT) scans. We conducted the cytotoxicity test by placing the materials subcutaneously and peri/intramuscularly. Days 30 and 90 were crucial for our research. On those days, we harvested the implants, kidneys and liver to search for any toxic deposits. The biomaterial’s uniformity, color and texture remained unaltered despite being in intimate contact with the tissue. Although a slight inflammatory response was observed in the placement location, we observed an improved outcome of the interaction between the material and its insertion area. There were no notable discoveries in the liver and kidneys. According to the obtained results, the biomaterials did not produce any clinical changes nor specific irritation during the research, demonstrating that they are biocompatible with biological tissues.

## 1. Introduction

The therapeutic efficacy of modern regenerative biomaterials relies on their durability and biological compatibility [1,2,3,4]. The latest development in biomaterial science is the integration of dental materials. Their compatibility and chemical resistance ensure successful therapy for a variety of disorders. Some of the newest substances contribute to bone rehabilitation and formation, as well as optimizing cell division and osteoblast proliferation [5,6,7,8].

Evaluating the biocompatibility of products that come into direct contact with normal tissues is vital for determining the degree of host–graft tolerance [9,10,11]. During the development of biomaterials, it is necessary to consider not only the durability, aesthetics or functionality of the material but also its suitability for biological use. The purpose of biomedical products is to provide optimal connections for the treatment, repair or replacement of affected tissues [1,12,13,14].

Cytotoxicity assays are crucial for evaluating products intended for use on animals or humans. To confirm the biocompatibility of a biomaterial, it is essential to understand the biological system in which the material will be used. Both in vitro and in vivo experiments are necessary to gain this understanding, though they cannot determine material compatibility with absolute certainty [15,16]. Some components may cause acute toxicity by accumulating in renal or hepatic cells.

Bis-GMA (bisphenol A-glycidyl methacrylate) is a resin that is used in dentistry due to its excellent mechanical properties [17,18,19,20]. When replaced with Urethane dimethacrylate (UDMA) monomer, the material’s durability, flexibility and polymerization shrinkage improve [21,22,23,24]. PEG-400 (polyethylene glycol 400) is a synthetic material known for its minimal toxicity [25,26]. Bioglasses are employed for their significant interactions with tissues, promoting bone development and angiogenesis [27,28,29].

The extensive use of cement extends to the field of orthopedics, particularly with PMMA (polymethyl methacrylate), which is widely employed for implant fixation or occasionally as a void filler. Predominantly used in hip and knee arthroplasty, PMMA relies on a mechanical interlock between the irregular bone surface and the prosthesis. However, a complication associated with this type of cement is bone cement implantation syndrome. Patients with this condition present with hypoxia, systemic hypotension, pulmonary hypertension, cardiac arrhythmias, loss of consciousness, and potentially cardiac arrest [30]. The onset time varies but typically occurs intraoperatively, either during cementation or prosthesis insertion. The causes of this syndrome are multifactorial and include a monomer-mediated model, embolus-mediated model, mechanical effects with high intramedullary pressure exceeding 300 mmHg during implant positioning, histamine release, hypersensitivity and complement activation [30].

Studies have shown that various implants can induce a local inflammatory response, which may sometimes lead to systemic complications, regardless of the implant’s biocompatibility [31]. Composite cements are formed from a polymer matrix with varying filler percentages [13,32]. Toxic activity and biocompatibility can be effectively managed through strict regulation of their structure [12,33].

Cements exhibit modest toxicity in the early stages, but this significantly decreases over time [34]. Some attribute this reduction to the non-polymerized constituents in the air-inhibited layer [35,36]. Using a living organism allows for complex interactions between the material and the biological system that cannot be replicated in ‘in vitro’ studies [37]. 

There have been studies where biocompatibility was evaluated using the tolerance test by subcutaneous and peri/intramuscular placement of composite cements [15,34,38,39,40,41]. The *novelty* of this work relies in establishing a new method of implanting biomaterials.

In *this study*, biocompatibility was evaluated using the tolerance test by subcutaneous and peri/intramuscular placement of two composite cements in rats. Biocompatibility was assessed by examining the implantation site (macroscopic analysis, histological analysis, CT scans), liver and kidney tissues, and clinical condition over a 90-day period. The animals were monitored through clinical observations of their appearance, behavior in the cage and handling.

## 2. Materials and Methods

### 2.1. Preparation of Biomaterials

The samples used in the study have a 25% weight. organic matrix (Bis-GMA; UDMA; HEMA; TEGDMA) and an 65% weight. bioactive salinized inorganic filler (hydroxyapatite, silica, barium glass and fluor aluminosilicate glass) [42]. The experimental cementing materials C1 and C2 were obtained by dispersing particles in the organic phase. The samples were initiated by photopolymerization, with camphorquinone (0.5% relative to the liquid mixture)/amine (1%) as the initiator/activator, using an O-Star Curing Light lamp (Guilin Woodpecker Medical Instrument, Co., Ltd., Guilin, China) for 20 s. The composition of both products is illustrated in Table 1.

**Figure 1:**
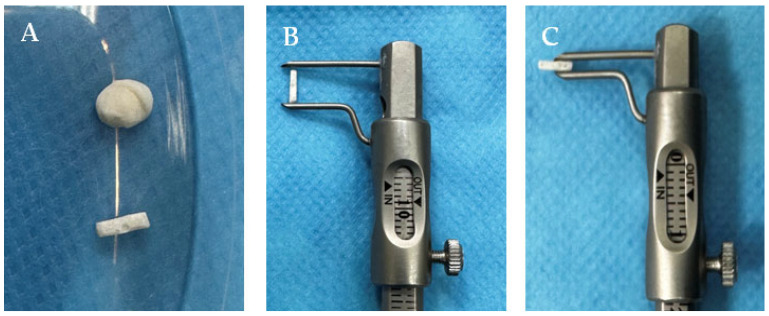
Biomaterial Cement C1. Biomaterial before and after polishing (**A**); 6.00 mm in height (**B**) and 1.40 mm in depth (**C**).

All biomaterials were polymerized prior to the procedure. Before implantation, the bioproducts were manually polished with a dental miller (Techno Med, Italia Group, Italy) from their initial round bead form to ensure that they perfectly matched the syringe’s dimensions. At the end of the process, the biomaterials measured 6.00 mm in length and 1.40 mm in width (Figure 1). The biomaterials were sterilized to eliminate microbial life forms by autoclaving them for 10 min at 105 °C in a Trade Raypa Steam Sterilizer (R. Espinar, S.L., Barcelona, Spania, Model AE-75 Dry).

**Figure 2:**
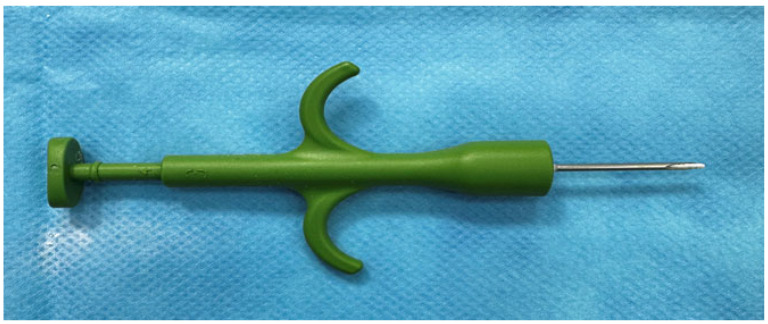
Pet microchipping sterile syringe used for implantation. The needle dimensions are as follows: E.D. (external diameter: 1.80–1.85 mm; I.D. (internal diameter: 1.45–1.50 mm); needle length: 3.00 cm).

The device we used for implantation was a pet microchipping sterile syringe (Figure 2) (Crotag International, Aldo Security, Tierchip Dasmann, Soartech LDT, Ilfov, Romania). The product according to standards [43,44]. The syringe did not require sterilization because it was already sterile.

### 2.2. Ethics Statement

The experiment was carried out at the Faculty of Veterinary Medicine’s Laboratory Animal Breeding and Usage Facility in Cluj-Napoca, Romania. The animals were sourced from the Experimental Medicine Center at the University of Medicine and Pharmacy Iuliu Hațieganu.

In compliance with standards [43,44], the rats were maintained under specific conditions, including a temperature of 23 °C, 55% humidity, and a 12 h light/dark cycle. The experiment received approval from the Bioethics Committee of the University of Agricultural Sciences and Veterinary Medicine Cluj-Napoca (approval no. 352/12.12.2022) and was authorized by the Sanitary-Veterinary and Food Safety Department of Cluj-Napoca (project authorization no. 374/04.07.2023).

The biocompatibility study was conducted in accordance laws on animal welfare [9,43,44,45].

### 2.3. Clinical Evaluation

Each rat was examined individually from day 0 to day 90, both inside and outside the cage. The inspections included body condition, eyes, limbs, fur, tail, and color. Handling was monitored to observe movements and body tone for any limitations or vocalizations. Additionally, various auditory, olfactory and visual stimuli were tested to assess neurological function [41].

### 2.4. Anesthesia

To make the procedure less painful, we utilized an induction box for rats. This is a small and transparent confinement area with a supply of anesthetic gas, Isoflurane (Isothesia 250 mL, Omegavet, Bucuresti, Romania) in our case, that we used to induce and maintain anesthesia during the procedure. The animals in use were placed in the box until they were anesthetized, and then the insertion was performed. It took around 7 s to place the biomaterial and after that the rats were provided with supplemental oxygen to avoid hypoxia and improve recovery [46].

### 2.5. Study Design

In the present study, as a biological resource, we used 8 adult female rats. The laboratory rats, at around 300 g in weight and 8 weeks old, belonged to the Muridae family, the Wistar–Lewis line [47]. This variety was chosen for its special features, such as greater obedience, excellent adaptability, and reduced vulnerability to infections [48,49]. The animals were divided into two groups of 4 rats each, one group for each tested biomaterial. Each group was further divided into two subgroups of two rats each, one for testing the biomaterial implanted subcutaneously and one for testing the biomaterial implanted intramuscularly.

On days 0, 10, 30, and 90, CT scans were performed. On days 30 and 90, tissue samples were taken for histopathological analysis. 

The animals were painlessly sacrificed through cervical dislocation while under general anesthesia, in conformity with international protocols, on day 30 and 90 of the research.

The research study was performed in the Faculty of Veterinary Medicine’s Establishment for breeding and use of laboratory animals in Cluj-Napoca, Romania, after the animals were obtained from the Experimental Medicine Center at the University of Medicine and Pharmacy Iuliu Hatieganu. 

### 2.6. Biomaterial Implantation

#### 2.6.1. Subcutaneous

The subcutaneous inoculation of polymerized cylindrical biomaterials was performed in the back area, interscapular, in the projection area of the first thoracic vertebrae using the inoculation syringe. The location was chosen considering that rats have a harder time accessing that area and would be unlikely to scratch or bite it. Moreover, it is easily kept under observation. The area was disinfected with 70% alcohol and the syringe was loaded with the polymerized biomaterial. Skin was lifted between the index and thumb, forming a skinfold, and the needle was inserted at a 45-degree angle. After insertion of the needle, the biomaterial was implanted by pushing the syringe plunge and advancing it into the tissue. All individuals adapted to the subcutaneous injection smoothly. Considering that the external diameter of the syringe is 1.8 mm, no suture was needed when the needle was withdrawn, the closure of the implantation site being ensured by the elasticity of the skin. The clinical status and behavior did not change.

#### 2.6.2. Intramuscular/Perimuscular

Peri/intramuscular inoculation of polymerized cylindrical biomaterials was performed in the right hind limb of each rat at the level of the biceps femoris muscle, using the inoculation syringe. The site was chosen based on the abundance of muscles. The implant was inserted as deeply as feasible into the muscles using a similar technique to those used in the presented reviews. Due to the needle’s modest diameter, the skin, fascia, and muscles did not require sutures after implantation. The procedure did not cause apparent mobility limitation. The clinical state and behavior did not alter.

### 2.7. Computed Tomography Scans

Helical CT scanning of the whole body was performed using a Siemens CT Somatom Scope machine with 16 channels. The scan was conducted with the patient in sterno-abdominal recumbency. The patient was fully sedated using inhalator anesthesia (Isothesia, Baia-Mare, Romania).

Body images were obtained in the axial plane using a 512 × 512 matrix, narrow windows (WW: 120, WL: 40), 3 mm slice thickness and a pitch of 3 mm. Multiplanar image reconstruction of the body was performed using soft tissue and bone window reconstruction at a slice thickness of 0.6 mm.

### 2.8. Histopathological Examination

Tissue samples from subcutaneous and peri/intramuscular inoculation, liver, and kidneys were taken at 30 and 90 days. They were then preserved for 24 h in 10% buffered neutral formaldehyde. After fixation, the tissues underwent conventional histopathology processing. The paraffin-embedded tissues were sliced into 2 μm thick sections and stained with Hematoxylin and Eosin (H AND E) stains for evaluation. The slides were examined by two pathologists using an Olympus BX41 microscope. The photographs were obtained with an Olympus UC 30 digital camera and examined using Olympus Stream Basic, a specialized capture and analysis program. On days 30 and 90 of the research, the materials were assessed and compared. Furthermore, liver and kidney samples were harvested to determine whether the usage of the material causes any form of acute toxicity.

The biomaterials were removed prior to tissue processing since they were too dense to be sliced.

## 3. Results

### 3.1. Clinical Evaluation 

Throughout the 90 days, the rats’ appearance did not change. We inspected the body’s shape, eyes and limbs. No alteration was seen. Fur, tail and color remained unchanged. When animal was removed from their cage to examine their responsiveness to handling, including motions and body tone, there were no vocalizations or movement impairments. A positive response to auditory, olfactory and visual stimuli was seen. When animals were taken out of their home cages, they were interested in investigating the new environment.

### 3.2. Postoperative Care

#### 3.2.1. Subcutaneous

The operation was painless, and the animals did not required analgesics based on the clinical examination. The animals were inspected on regular intervals, and there was no indication of suffering.

#### 3.2.2. Intramuscular/Perimuscular

For this group, we utilized Buprenorphine (1 mg/kg Bupaq, Biotur, Teleorman, Romania) subcutaneously in the first day. No further analgesia was provided due to a good clinical evolution. The animals showed no signs of limping, and no extra attention was paid to the region. The justification for utilizing analgesia was based on the fact that the muscles have multiple pain receptors. Subsequently, there were no signs of pain.

### 3.3. Biomaterial Implantation

#### 3.3.1. Subcutaneous

All individuals tolerated the subcutaneous biomaterial good. There was no change in the behavior. During the physical examination, the implant was barely perceptible when touched; solidly attached, with no volume, consistency, or color alterations; and non-painful.

**Figure 3:**
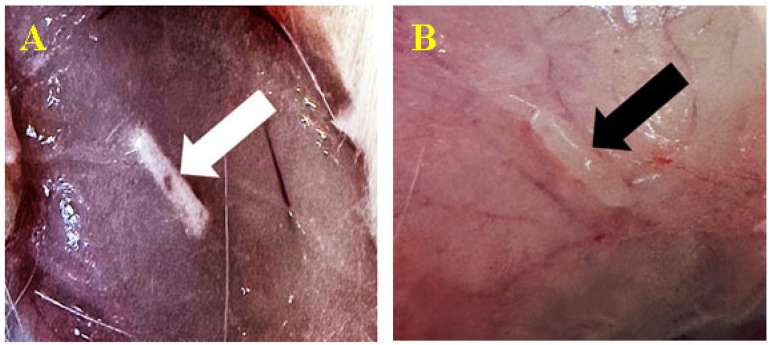
Biomaterial implantation subcutaneously. Biomaterial C1(**A**-white arrow) and biomaterial C2 (**B**-black arrow) at 30 days after inoculation.

After 30 days, we removed the skin to analyze the subcutaneous tissue with the biomaterial (Figure 3). The whole area of the subcutaneous reaction was harvested. We identified the material encapsulation resulting from developing fibro-vascular connective tissue. The tissue develops as a fatty capsule from the subcutaneous connective cells, which surrounds the product while preserving its original state.

**Figure 4:**
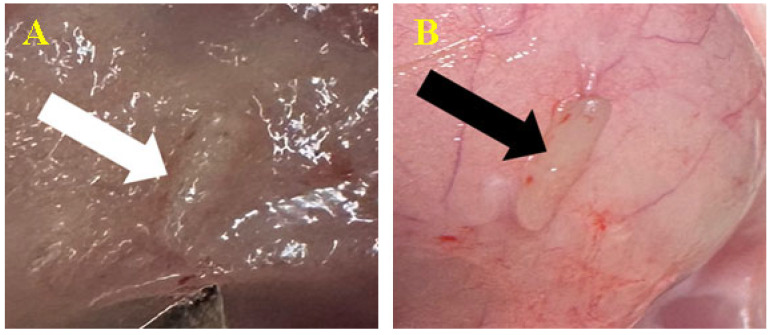
Biomaterial implantation (subcutaneous). Biomaterial C1 (**A**-white arrow) and biomaterial C2 (**B**-black arrow) at 90 days after inoculation.

On day 90, the same processes occurred, but with more pronounced elements. No apoptosis or rejection processes were seen (Figure 4).

#### 3.3.2. Peri/Intramuscular 

The procedure was completed with no complications. The biomaterial did not induce noticeable mobility limitation or scar tissue. During the inspection, the implant was undetectable, with no volume, consistency or color changes, and it was painless. There was no evidence of limping, and no special care was given to the area.

**Figure 5:**
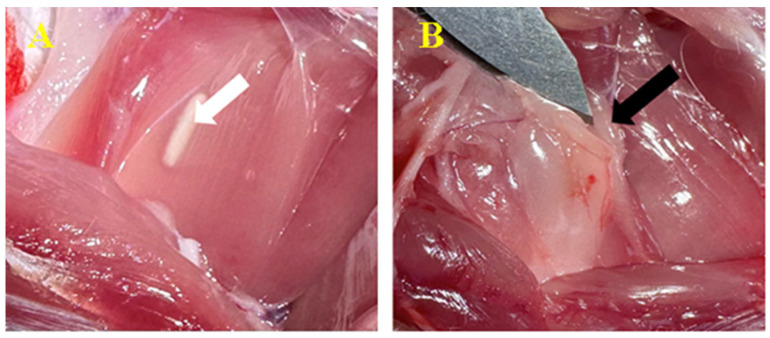
Biomaterial implantation (intramuscular). Biomaterial C1 (**A**-white arrow) and biomaterial C2 (**B**-black arrow) at 30 days after inoculation.

**Figure 6:**
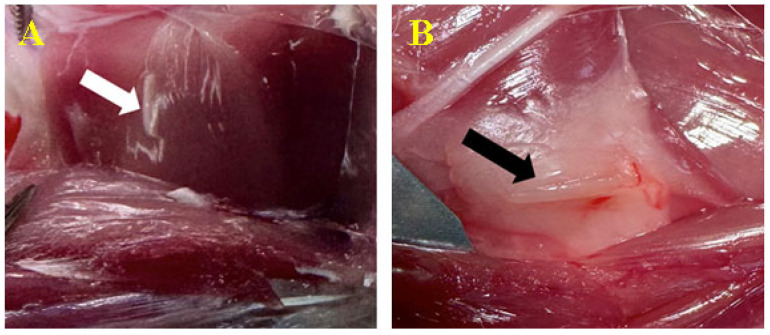
Biomaterial implantation (intramuscular). Biomaterial C1 (**A**-white arrow) and biomaterial C2 (**B**-black arrow) at 90 days after inoculation.

To access the implant, the skin needed to be removed and the intermuscular area examined. Deep in the intermuscular fascicles, the biomaterial was revealed to be surrounded by a layer of fatty-like connective tissue capsule with minimal vascular reactivity induced by neoformation vessels (Figure 5). No rejection processes were seen. Day 90 indicated the same activity, but with a more visible tissue response (Figure 6).

### 3.4. CT Image Findings

**Figure 7:**
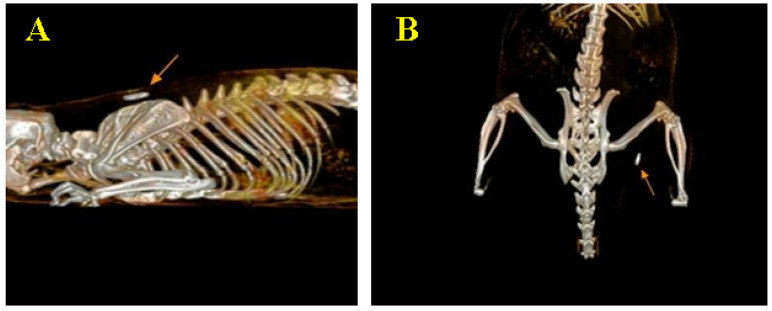
A 3D reconstruction of the images, indicating their position: (**A**)—subcutaneous implantation at cervical dorsal interscapulum level (marked with the orange arrow) and (**B**)—intramuscular implantation at the level of the femoral biceps muscle in the right posterior limb (marked with the orange arrow).

The 3D reconstruction of the images was performed to verify the correct implantation of the two materials, respectively, at the subcutaneous level in the dorsal cervical interscapulum region and at the level of the biceps femoris muscle of the right posterior limb (Figure 7). Measurements were also performed to determine the dimensions of the materials, whose standard dimensions established as a result of the measurements were 6 mm on the length axis and 1.4 mm on the width axis.

Scans of the whole organism were performed on the day of implantation of the materials, after 10 days, 30 days and 90 days after implantation. Each time, the entire body was scanned in case the materials had migrated from the implantation site. No migration of the materials was observed, though we did observe changes in the position of the longitudinal axis of the materials. 

At each scan, the materials were measured to see if there was any resorption of them, but no changes in dimensions were observed; until day 90, both materials maintained the same dimensions observed at the beginning of the study, i.e., 6 mm/1.4 mm.

The Hounsfield units of the materials were also measured. Material C1 has an average of Hounsfield units of 2350 HF and material C2 has an average of 2016 HF, so material C1 has a higher radiodensity than material C2, but both have a density specific to dense bones [45,50].

**Figure 8:**
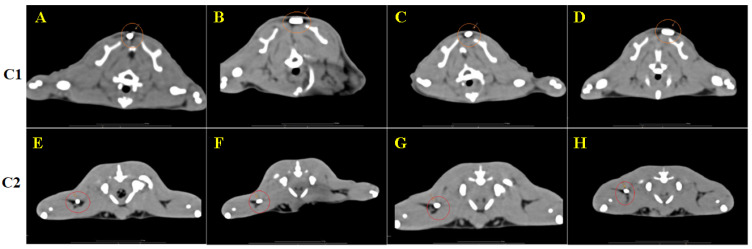
Axial section at the level of the subcutaneously implanted material (**A**–**D**) at 0, 10, 30 and 90 days; the area with the implanted material is marked with an orange circle; axial section at the level of the intramuscularly implanted material (**E**–**H**) at 0, 10, 30 and 90 days; the area with the implanted material is marked with a red circle.

The CT images indicated an inflammatory reaction in the area of implantation of the materials both subcutaneously and intramuscularly, a reaction that no longer appeared after the 10-day exposure; otherwise, no other remarkable changes were observed in the scans performed 30 days and 90 days after implantation (Figure 8). 

### 3.5. Histopathological Findings

**Figure 9:**
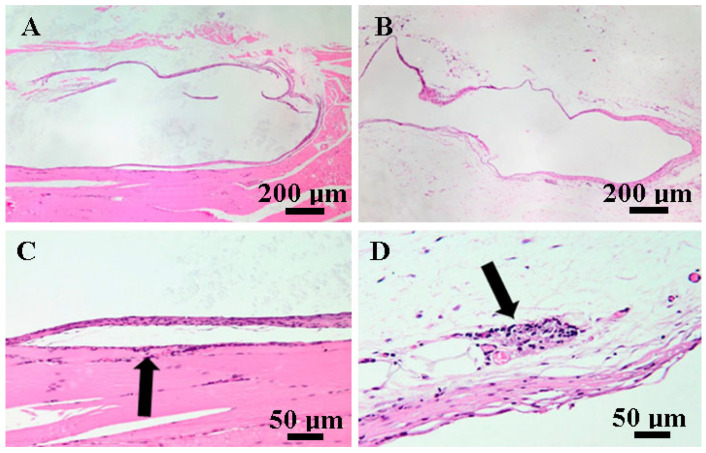
Photomicrographs of muscle (**A**,**C**) and subcutaneous tissue (**B**,**D**) on day 30. (**A**,**B**) The biomaterials are encased by fibrous tissue composed of plump fibroblasts and mature collagen fibers, H AND E, bar = 200 μm. (**C**,**D**). Within the capsule, there is a mild inflammatory infiltrate consisting of macrophages, occasional heterophils, lymphocytes, and rare hemosiderin-laden macrophages (siderophages) (black arrow), H AND E, bar = 50 μm.

On day 30, rat tissue specimens from subcutaneous tissue and muscle treated with C1 and C2 biomaterial showed similar histological changes. A fibrous capsule isolated the biomaterials (Figure 9A,B). The capsules were mostly made up of fibroblasts and mature collagen fibers, with the occasional presence of inflammatory cells such as macrophages, siderophages, rare heterophils and lymphocytes both within the capsule and perivascular. Multifocally, the proliferation of new blood vessels was noted. The adjacent tissues exhibited normal characteristics.

**Figure 10:**
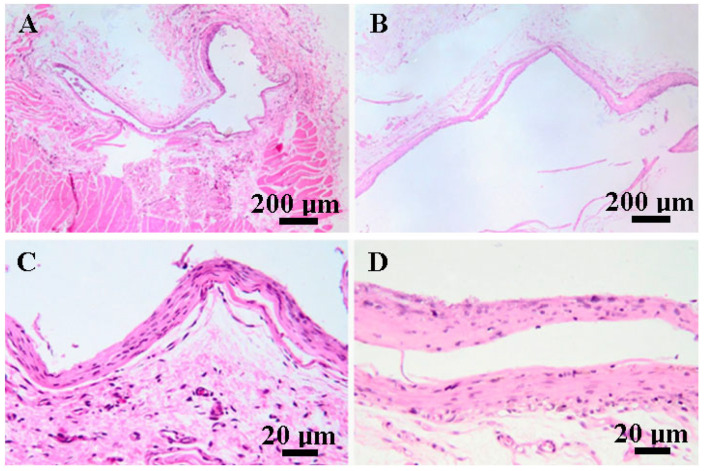
Photomicrographs of muscle (**A**,**C**) and adipose tissue (**B**,**D**) on day 90. (**A**,**B**) the biomaterials are delimited by a fibrous capsule, composed of fibroblasts and mature collagen fibers, H AND E, bar = 200 μm. (**C**,**D**). Rare inflammatory cells represented by macrophages, lymphocytes, and plasma cells are present in the adjacent tissue, H AND E, bar = 20 μm.

On day 90, rat tissue samples treated with C1 and C2 exhibited similar transformations to those seen on day 30. The biomaterials were still surrounded by fibroblasts and mature collagen fibers (fibrous tissue) (Figure 9A,B), and minimal inflammatory infiltrate, represented by macrophages, lymphocytes and plasma cells, was observed within the adjacent tissue (Figure 10C,D). There were no significant changes to the muscular or subcutaneous tissues.

**Figure 11:**
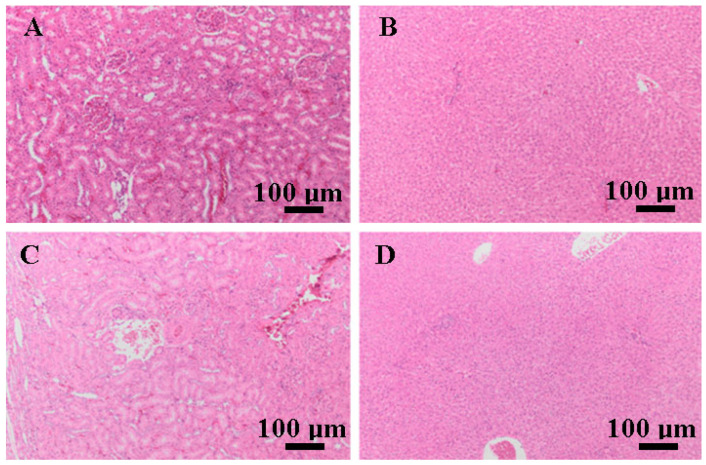
Photomicrographs of kidney (**A**,**C**) and liver (**B**,**D**) on day 30 (**A**,**C**) and 90 (**B**,**D**) H AND E, bar = 100 μm, without any notable findings identified.

During the study, hepatic and renal tissues were collected to assess any type of cell toxicity. The histopathological study revealed remarkable findings. The organism encapsulated the biomaterial without expressing any response to it in the organs (Figure 11).

## 4. Discussions

Research on biocompatibility, such as that conducted by Ivanov et al. and El Reash et al., shows that biocompatibility and the local inflammatory response are highly dependent on each other. Moreover, the degree of local response and the resulting fibrous tissue might influence the bio-acceptance [51,52] Our results showed very little evidence of an inflammatory infiltration, and the surrounding tissues maintained their functions normally. In light of these considerations, significant efforts have been made to develop products with fewer hazardous biological implications.

Investigating behavior is critical for the early detection of any product-related side effects. When assessing rat behavior, several variables must be considered, including appearance, neurological state, mobility, reflexes, and motion [42]. While some parameters are subjective, they still highlight important traits that should be monitored carefully. 

Drug administration was used to control pain (in our case, buprenorphine, for the intramuscular implantation batch). The animals continued to act normally, though. The rats’ usual food and drink consumption indicates that the composite cement implantation did not cause any harm to them. These data support the credibility and accuracy of research findings in addition to guaranteeing the ethical treatment of rodents [53].

This study has a contribution of originality through the use of a microchipping syringe as a tool for insertion of the implant. The pet microchipping device allowed us to significantly reduce scar tissue during healing compared to the standard biomaterial insertion method. The conventional implantation of the biomaterial involves cutting through skin and muscle, which might result in a lot of scar tissue. Due to the large amount of local scar tissue in other studies, they cannot adequately reflect the body’s reaction to biomaterials. Based on previous research, one may conclude that the inflammatory changes observed in the surrounding tissues may be mostly caused by tissue damage incurred during surgery [54,55].

Perforation wounds are the most common kind of wounds caused by a syringe during an injection. Suturing is frequently not required for puncture wounds (less than 5 mm), unless the incision is in a high-tension location. Moreover, research shows that sutures are frequently not necessary for small needle punctures, such as those made using a 2 mm needle [56]. Due to the small size of the wound and the elected site, we chose not to use any suture material on our subjects. One of the main benefits that we employed in our research was this non-invasive strategy.

The implantation of a foreign product in rats causes a variety of biological responses in their muscular tissues. The organism immediately responds by going into an acute inflammatory reaction. First to come are neutrophils and macrophages. Their duty is to remove the debris. Later, granulation tissue starts to develop, marked by the emergence of new blood vessels, immune cells and fibroblasts. These structures help to promote repair and separate the foreign substance from the surroundings. The initial stage of biomaterial encapsulation is dominated by macrophages that merge to produce giant cells, which are considered characteristic of a foreign body reaction. Histological examination revealed a mild foreign body reaction, which is normal for this phase [57,58]. By day 90 of the trial, the reaction had decreased in extent, and the cells around the capsule showed typical features, without any necrosis or apoptosis. There were no signs of muscular atrophy or tissue remodeling either. These polymers exhibit biocompatibility, since we did not see any indications of rejection or a significant inflammatory reaction. Both in vivo and in vitro tests showed no signs of local cytotoxicity from these products [13].

The cement was inserted instantly and the anesthesia duration was shortened, which had a lesser negative impact on the body. Additionally, anxiety as well as stress were reduced.

Animals can become stressed and experience pain as a result of handling and injections, which may have an impact on the results of experiments, particularly those that include discomfort and anxiety. There are a number of significant distinctions between inhalational and intraperitoneal (IP) anesthesia in rats, including administration, physiological consequences, and applicability for different types of experiments [59,60]. Using inhalatory anesthesia was the best option in this investigation. The utilization of a chamber and then a mask simplified the induction and maintenance of anesthesia. Using this method, we could initiate rapid induction and the patient recovered faster.

The implant did not interfere with mobility, feeding or other clinical behavior. The fact that the rodents could walk unhindered and exhibit normal activities suggests that the implants were accepted. Following implantation, their body weight and eating patterns were consistent [61]. By comparing histology data to clinical evaluation, we established an encouraging confirmation of body acceptance of the biomaterial.

The examination and evaluation regarding intramuscular and subcutaneous tolerances permits us to conclude that the materials, in both the form and structure provided, could be effortlessly inserted in favorable circumstances. After 90 days in direct contact with the tissue, the biomaterial remained unchanged. There were no challenges or unusual postoperative incidents. The products remained biocompatible within biological parameters. They only appeared in new connective tissue, which is specific to the healing process. 

Local toxicity results from the chemical interaction between a toxic substance and biologically relevant molecules. Meanwhile, tissue compatibility may depend on factors beyond just the material’s toxicity [33].

There was no evidence of composite material deterioration after 90 days of implantation. Consequently, the organism was not exposed to any toxins. We have provided the liver and kidneys’ histopathology examinations as evidence. This is essential for determining the stability over the course of time and any possible impacts on nearby structures.

The primary outcomes of this experiment revolve around assessing the capacity of dental cement materials to stimulate bone formation in living organisms.

The toxic potential of materials and devices significantly depends on the filterability and toxicity of the soluble components present in the material composition. The number of compounds released into the environment is related to the surface area and thickness of the extracted compound, and also to temperatures approximating the normal human body temperature.

Their impact on health has demonstrated that these leftovers could potentially be cytotoxic. By conforming to established polymerization methods, making sure that curing periods were sufficient, and employing the right curing lamps, we were able to reduce the amount of residual monomers [62,63].

The chemistry, size and degradation pattern of the materials significantly affect the biological response.

In vivo evaluation of nonspecific tissue reactions to biomaterials generally involves histological investigations after the material is implanted in animal tissue [64]. After a short implantation period of one or two weeks, inflammation around the implant is visible, while after a longer period, the nature and amount of connective tissue encapsulation can be assessed [65].

Studies on subcutaneous tissue and muscle treated with C1 and C2 biomaterial cements containing the same bis-GMA proportion showed similar histological changes. Although blood cells are not directly influenced by monomeric Bis-GMA, this monomer can lead to the overexpression of proinflammatory cytokines due to its cell toxicity [66].

Bis-GMA is the most viscous and least flexible monomer due to its strong intramolecular hydrogen bonds with its hydroxyl groups and the presence of rigid aromatic nuclei in its structure [67]. 

Another critical factor influencing the leaching of residual monomers is the nature and molecular size of the monomers in the resin. Smaller molecules tend to leach more quickly than larger ones and can be extracted in significantly larger amounts. For instance, TEDGMA, a low-molecular-weight monomer, exhibits greater mobility and elutes faster than larger molecules like Bis-GMA. This greater mobility might explain the inflammatory response observed in this study with resins containing Bis-GMA. Additionally, Bis-GMA’s poor water solubility makes it difficult to dissolve over time, further affecting its leaching behavior.

Ultimately, understanding the local interactions of materials with tissues, including bone, is crucial. In vitro and skin tests can provide conditions similar to in vivo tests with a highly controlled environment, lower costs, and faster procedures [65]. These equivalents have an interesting perspective, but experiences with biomaterials remain very limited [68,69,70].

A limitation of the research is the reduced number of subjects. Given that the skin can heal minor wounds quickly and since the syringe’s diameter was under 2 mm, we did not employ any control groups. For future research, we will consider expanding the number of tested rodents and also investigating a control batch.

## 5. Conclusions

The current study provides a unique contribution by using a microchipping syringe as a tool for material placement. 

The cements were anchored and encapsulated in a fatty-like membrane, becoming thinner or thicker depending on the time they spent in their host. The biomaterials did not produce any clinical changes nor specific irritation during the research.

According to the CT scans, no changes in dimensions were detected for both cements.

The histopathology observations demonstrate suitable compatibility without negative consequences. No signs of cytotoxicity or rejection were noticed. The in vivo implantation model employed in our studies did confirm the in vitro findings demonstrating feasible biocompatibility.

Given all of the information supplied, we may conclude that the biomaterials are biocompatible with biological tissues such as the one tested in this research.

## Figures and Tables

**Table 1:** Experimental cement composition.

Materials	Manufacturer	Matrix Monomers	Total Content Filler
Light-curing hybrid cement composite (C1)	UBB-ICCRR, Cluj-Napoca, Romania	Bis-GMA ^1^; UDMA; HEMA TEGDMA ^3^;	65 weight%,—HA ^2^ (particle size 0.01–0.06 μm and 5–8 nm); silica, barium glass (BaO) (particle size 0.01–0.035 μm and 2–6 nm); quartz
Light-curing hybrid cement composite (C2)	UBB-ICCRR, Cluj-Napoca, Romania	Bis-GMA ^1^; UDMA; TEGDMA ^3^;	65 weight%, HA ^2^ (particle size 0.01–60 μm and 5–8 nm); silica, glass filler (with BaF2) (particle size 2–6 nm); fluoroaluminosilicate glass (0.04–0.50 μm)

^1^ Bis-GMA—2,2-bis(3-(2′-hydroxy-3′methacryloyl-oxypropoxy)phenyl) propane; ^2^ HA—hydroxyapatite; glasses (synthesized in UBB-ICCRR laboratory, Cluj-Napoca, Romania); ^3^ TEGDMA—triethyleneglycol-dimethacrylate (Aldrich, Steinheim, Germany); DMAEM-2-dimethyl(aminoethyl)methacrylate (Aldrich, Steinheim, Germany), Cq—camphorquinone (Aldrich, Steinheim, Germany).

## Data Availability

The original contributions presented in the study are included in the article, further inquiries can be directed to the corresponding author.
